# Tourists’ Perceptions of the Free-Roaming Dog Population in Samoa

**DOI:** 10.3390/ani4040599

**Published:** 2014-09-29

**Authors:** Magnus Beckman, Kate E. Hill, Mark J. Farnworth, Charlotte F. Bolwell, Janis Bridges, Els Acke

**Affiliations:** 1 Department of Clinical Sciences, Faculty of Veterinary Medicine and Domestic Animal Sciences, Swedish University of Agricultural Sciences, Box 7054, Uppsala 75007, Sweden; E-Mail: magnus_beckman@hotmail.com; 2 Institute of Veterinary, Animal and Biomedical Sciences, Massey University, Private Bag 11222, Palmerston North 4442, New Zealand; E-Mails: k.hill@massey.ac.nz (K.E.H.); c.bolwell@massey.ac.nz (C.F.B); j.p.bridges@massey.ac.nz (J.B.); e.acke@massey.ac.nz (E.A.); 3 Animal Welfare and Biodiversity Research Group, Department of Natural Sciences, Unitec Institute of Technology, Private Bag 92025, Auckland 1025, New Zealand

**Keywords:** animal welfare, developing nation, dog, free-roaming, population management, tourism

## Abstract

**Simple Summary:**

For travelers, the way in which people in other nations interact with animals may be different to that in their home nation. This research explores how the treatment of dogs impacted upon the holiday experiences of tourists visiting a developing island nation. In general, and where tourists encountered dogs, their treatment was perceived as less positive than in their home country and had a negative impact upon the holiday experience. Although it is important to recognize that the local population will have a different worldview, tourists felt that the dog population required more effective management and were most supportive of techniques that were non-lethal and humane.

**Abstract:**

A study was undertaken to establish how visiting tourists to Samoa perceived free-roaming dogs (*Canis familiaris*) and their management, additionally some factors that influence their perceptions were assessed. Questionnaires were administered to 281 tourists across Samoa over 5 weeks. Free-roaming dogs were seen by 98.2% (*n* = 269/274) of respondents, with 64.9% (*n* = 137/211) reporting that their presence had a negative effect on overall holiday experience. Respondents staying in the Apia (capital city) area were more likely to consider dogs a problem (*p* < 0.0001), and there was a significant association between whether the respondent owned a dog and if they thought dogs were a nuisance in Samoa (*p* < 0.003). Forty-four percent (20/89) of non-dog owners agreed that dogs were a nuisance compared to 22% (80/182) of dog owners. The majority felt that dogs required better control and management in Samoa (81%, *n* = 222) and that there were too many “stray” dogs (67.9%, *n* = 188). More respondents were negatively affected by the dogs’ presence (64.9%, 137/211), and felt that the dogs made their holiday worse, than respondents that felt the dogs’ presence improved their holiday experience (35.1%, 74/211). Most respondents stated that the dogs had a low impact (one to three; 68%, 187/275) on their stay in Samoa, whilst 24% (65/275) and 8% (23/275) stated they had a medium or high impact, respectively, on their stay. Respondents showed strong support for humane population management. Free-roaming dogs present a complex problem for Samoa and for its tourism industry in particular. The findings of this study further support the need for more discussion and action about the provision of veterinary services and population management for dogs in Samoa. It also provides information complementing an earlier study of the attitudes of local Samoans.

## 1. Introduction

Tourism is the major industry in many island nations including Western Samoa (Samoa) where 134,700 visitors arrived in 2012, mostly from New Zealand or Australia [1]. Cultural differences and expectations will often either positively or negatively affect overall holiday experience, [2] which, in turn, may alter the likelihood of repeat visits. The literature primarily focuses on tourist-animal interactions that are actively sought out and that provide positive experiences with wild or semi-wild animals [3,4]. Such interactions have the potential to significantly benefit the tourism industry but also cause ecological damage [5]. To date little exploration has been made of incidental interactions between tourists and free-roaming dogs. For many tourists, expectations concerning treatment of dogs will likely be informed by personal experiences and expectations from their own country. It is possible that a mismatch between tourist attitudes and those of the country being visited may occur. These mismatches in attitudes towards dogs may present a significant challenge to a developing tourism industry. Mismatches between promoted and actual experiences in animal-based tourism are likely to result in negative reporting of the holiday experience [6], and this is likely the case even when the interaction is incidental.

Evidence from Samoa suggests that a substantial number of households own a dog. Many of these dogs are free-roaming and unsterilized, and dog bites are relatively commonplace [7]. For primarily socio-economic and accessibility reasons, most Samoan dogs (72%) have never been provided with veterinary care [7]. Currently, veterinary care for companion animals in Samoa is only provided in the capital city by the Animal Protection Society (Apia, Samoa). Injuries to humans caused by dog bites are of concern with high numbers of dog bites reported in both Samoa and American Samoa [7,8]. As a consequence of bites and dogs being considered a nuisance, local residents may inflict physical harm upon the dogs [7]. The definition of a “free-roaming dog”, which includes both owned and un-owned individuals, is “one that is not currently under direct control or is not currently restricted by a physical barrier” [9]. The characteristics of a free-roaming dog population include no restraint (*i.e.*, no leash or fencing to confine the dog to a property), low average age [10] and a male-skewed sex ratio [11]. Such characteristics are often described in relation to dog populations in developing countries [10].

Free-roaming dog populations are linked to a host of problems that range from animal welfare to public health [12,13,14]. Major concerns identified globally, but with particularly high levels of report in developing countries, include the risk of dogs biting people, increased transfer of zoonotic disease [15], and negative impacts upon local wildlife [16]. Relatively little veterinary care or public education about dogs has, in part, led to negative welfare consequences for free-roaming dogs internationally including malnourishment, infectious and parasitic diseases, and injury due to traffic accidents, fighting or abuse from humans [12,14,17]. Concerns about animal welfare have been reported from various countries, including those with tourist areas where free-roaming dogs are present [18,19].

The perceptions of tourists and their interaction with Samoa’s free-roaming dog population, as well as the influence of the dogs on the holiday experience, have not been assessed. A mismatch between expected dog behavior based on tourist perception and prior experience in the home nation may put individuals at risk. Tourists’ opinions may also function to support dog welfare and population management initiatives which, given the local issues associated with free-roaming dogs, may provide Samoa with information which they may opt to use to inform dog management. The aims of the present study were to determine the potential negative (or positive) impact of dogs on the experiences of tourists in Samoa, and to establish attitudes of the tourists regarding management and control of the local dog population. The tourists’ demographic details, interactions with and perceptions of dogs, both in Samoa and in their countries of origin were also investigated.

## 2. Material and Methods

A questionnaire (Supplementary Material) concerning tourists’ attitudes and reactions towards dogs was distributed to adult tourists in Samoa during high season over a 5-week period (August and September) in 2012. Respondents had to have been in Samoa for a minimum of 2 days. All major tourist areas were included, those being Apia (including surroundings), the south coast of Upolu and the east and north coast of Savaii. The questionnaire was pre-tested with students and staff at Massey University (Palmerston North, New Zealand), and one veterinary surgeon (Magnus Beckman, the first author of this manuscript) was in charge of questionnaire distribution in Samoa. The project and questionnaire were screened by the Massey University Research Ethics Office, evaluated by peer review, and judged to be low risk. Consequently it was not evaluated by one of the University’s Human Ethics Committees (Palmerston North, New Zealand). The researchers were responsible for the ethical conduct of this research.

### 2.1. Sample

Individuals in the tourist areas were approached and asked if they were over 18 years of age, if they were from overseas and if they had been in Samoa for a minimum of two days. If these inclusion criteria were fulfilled each person was asked if they would participate in a short survey regarding dogs in Samoa. In addition respondents were given a written introduction to the survey and its goals. The questionnaire was delivered in public spaces and around tourist accommodations. In general, for crowded areas every third person was asked to participate, whilst in less crowded areas every other person was asked. In areas with sparse tourist presence every person was asked to participate. If a person declined to participate, the next person was asked until someone agreed to participate. Questionnaires were also supplied to hotels, resorts, and budget accommodations around Samoa. Of 50 tourist accommodation establishments approached, 35 agreed to surveys being left at the hotel reception, of these 23 also consented to their guests being approached for the purposes of an interview.

### 2.2. Questionnaire Design

A brief written introduction was given as part of the questionnaire, describing aims, consent and confidentiality. The questionnaire was in English, anonymous, and consisted of five sections. The first gathered the respondents’ demographic details; the second covered opinions on dogs and their management, both outside of and within Samoa, and used a Likert scale to gauge respondents’ levels of agreement (1 = absolutely agree; 5 = absolutely disagree) with a predetermined set of questions. Respondents could also indicate that they did not have an opinion by selecting a “don’t know” option. The third section gathered information on the respondents’ experiences with Samoan dogs and their behavior around dogs in Samoa. The fourth section addressed the free-roaming dog population’s impact on their overall holiday experience using a 1–10 rating scale where 1 was “no impact” and 10 was “very serious impact”. Respondents were also asked to indicate if their experience was generally positive or negative. Finally the last section assembled attitudes towards free-roaming dog population management using Likert scales as for section two. The respondents were able to make additional comments at the end of the questionnaire.

### 2.3. Data Analysis

Analyses of data were performed with Microsoft^®^ Excel 2002 and R version 2.15.1. All binary and categorical data were summarized as counts and percentages and continuous, non-parametric data were summarized as median and interquartile (IQR) range. For analysis, the duration of the respondents’ stay in Samoa was categorized into a binary variable (2–14 days; >14 days). Chi square tests and Fisher’s exact tests, for low cell frequencies, were used to compare binary and categorical variables of interest. These included gender, length of stay and dog ownership (respondent did or did not own a dog) with whether the respondents reported dogs were found to be a nuisance in Samoa, whether the dogs required better management in Samoa, whether violence towards dogs in Samoa was unacceptable, and if the respondents’ holidays had been affected by dogs in Samoa. Cramer’s *V* was calculated to show the effect size for the Chi square and Fisher’s exact tests, for contingency tables with more than 1 degree of freedom. Respondents’ answers to whether dogs were a problem in Samoa, if they had seen inappropriate behavior towards dogs and if dogs had affected their holiday experience were compared by region. After a Bonferroni correction was applied to adjust for multiple comparisons, the level for statistical significance was *p* < 0.004.

## 3. Results

A total of 281 questionnaires were collected from tourists in Samoa over the 5-week period of 2012 and analyzed in this study. The non-response rate was not able to be accurately determined, but the number of people who declined to participate was estimated to be 10% of those approached, and approximately 20% of 281 completed questionnaires were gathered from the 35 accommodation establishments where questionnaires were left at reception. The questions concerning hygiene habits with regard to dog contact (see Supplementary Material) are not the focus of this manuscript and are not accounted for further.

### 3.1. Respondent Demographics

Forty-six percent (128/279) of respondents were male. Four percent of the 281 respondents were aged 18–20, 47% were aged 21–40, 36% were 41–60, 12% were 61–80, and only 1% was 80+ years of age. The majority of respondents came from New Zealand (55.4%; 153/281) and Australia (24.3%; 67/281), and had been in Samoa for a median of 7 (IQR 4–10) days. Most (85% 245/280) respondents had been in Samoa between 2 and 14 days, and 14 respondents had been in Samoa for over 300 days; as they recorded themselves as not being from Samoa, they were included in the dataset. Overall, 90/279 respondents (32.3%) reported owning a dog, most of which were companions. Gender did not influence whether a respondent owned a dog (*p* = 0.24, Cramer’s *V* = −0.08). 

### 3.2. Opinions Regarding Dogs

**Figure 1:**
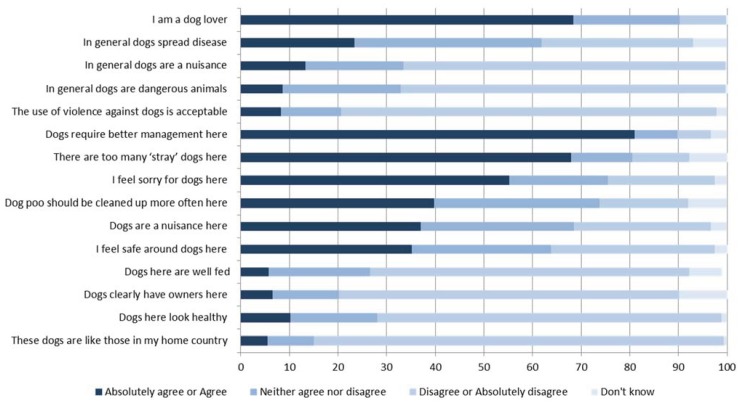
Opinions on dogs in general and in Samoa as stated by 281 respondents of a questionnaire on tourist attitudes, experiences and behavior around Samoan dogs. The percentage (%) of tourists’ responses for each category is shown on the y-axis and the questions on the x-axis.

The majority of respondents felt that dogs required better control and management in Samoa (Figure 1) and that there were too many “stray” dogs. There was a significant association between whether the respondent owned a dog and if they thought dogs were a nuisance in Samoa (*p* < 0.003, Cramer’s *V* = 0.23); 44% (20/89) of non-dog owners agreed that dogs were a nuisance compared to 22% (80/182) of dog owners. There was no effect of gender or length of stay on whether dogs were considered a nuisance in Samoa (*p* = 0.75, Cramer’s *V* = 0.08; *p* = 0.10, Cramer’s *V* = 0.10, respectively). There was no effect of gender (*p* = 0.92, Cramer’s *V* = 0.04), length of stay (*p* = 0.64, Cramer’s *V* = 0.10) or dog ownership (*p* = 0.88, Cramer’s *V* = 0.06) on whether respondents thought dogs required better management in Samoa. There was no effect of gender or dog ownership on whether respondents thought violence against dogs was unacceptable (*p* = 0.05, Cramer’s *V* = 0.16; *p* = 0.50, Cramer’s *V* = 0.09 respectively).

### 3.3. Experiences and Human Behavioral Responses towards Dogs

Most respondents had seen free-roaming dogs in Samoa, said they avoided contact with the dogs, and said they felt threatened by the dogs (Table 1). Some respondents reported witnessing inappropriate behavior against dogs (Table 1), which was defined as physical abuse (65.1%, 28/43), physical abuse and lack of care (18.6%, 8/43), or just lack of care (16.3%, 7/43).

**Table 1:** Experiences and behavioral responses towards dogs in Samoa stated by 281 responding tourists in Samoa in a questionnaire on attitudes, experiences, and behavior around Samoan dogs (N (%)).

Question	Yes, Always or Sometimes	No, Not at All	Total
Have you seen free roaming dogs in Samoa?	**269 (98.2)**	5 (1.8)	274
Do you avoid contact with dogs here?	**235 (86.4)**	37 (13.6)	272
Do you feel dogs are friendly here?	**210 (79.5)**	54 (20.5)	264
Do dogs here seem frightened and avoid human contact when approached?	**180 (66.7)**	90 (33.3)	270
Do you feed the dogs here?	67 (24.6)	**205 (75.4)**	272
Do dogs here beg for food?	**178 (66.2)**	91 (33.8)	269
Do you feel threatened when a dog approaches you here?	**171 (63.8)**	97 (36.2)	268
Have you witnessed inappropriate behavior against dogs in Samoa?	66 (25.2)	**196 (74.8)**	262

Numbers in bold indicate a majority of the respondents to a question. Total in table are the number of responses to a specific question.

### 3.4. Opinions on Population Management

**Figure 2:**
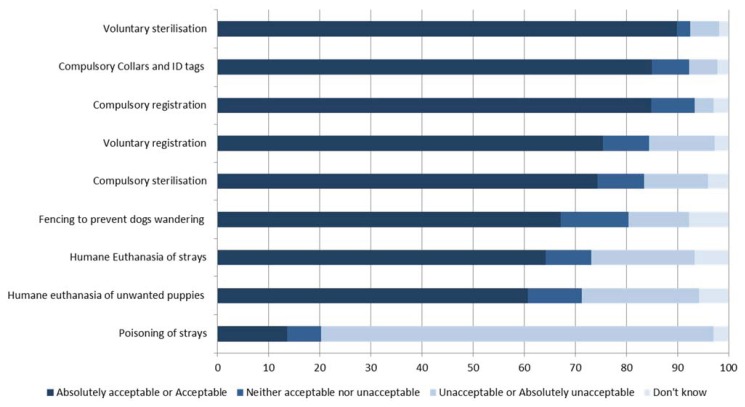
Methods for management of dog populations stated by 281 responding tourists in Samoa in a questionnaire on attitudes, experiences and behavior around Samoan dogs. The percentage (%) of tourists’ responses for each category is shown on the y-axis and the questions on the x-axis.

Of the methods for dog management mentioned in this study, the strongest support was earned by voluntary sterilization (Figure 2), closely followed by compulsory collars and ID tags and compulsory registration. The poisoning of strays was regarded as unacceptable for the vast majority of respondents.

### 3.5. Differences in Perception of Dogs Dependent on Location of Accommodation

**Figure 3:**
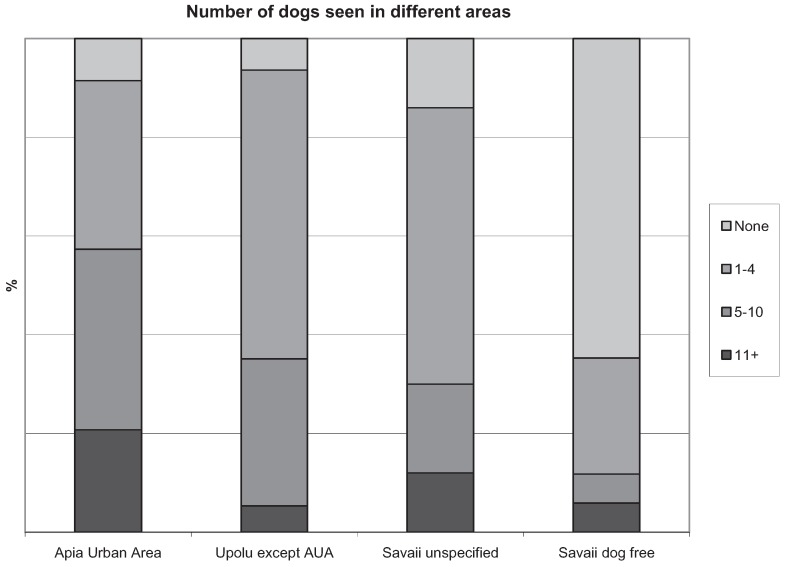
Percentage of respondents (243) who reported the number of dogs seen around the accommodation areas in Samoa, taken from the questionnaire on attitudes, experiences, and behavior around Samoan dogs.

In Apia, (75%, 60/80) as compared to the rest of Upolu (44%, 41/93), dogs were more commonly perceived as a problem (*p* < 0.001, Cramer’s *V* = 0.26). Inappropriate actions against dogs were more commonly seen in Apia (34%, 27/80) than the rest of Upolu (14%, 12/87) (*p* = 0.0038). There were no significant associations between holiday experience and different regions. Figure 3 illustrates the percentage of respondents who reported number of dogs seen in different areas.

### 3.6. Overall Experience

More respondents were negatively affected by the dogs’ presence (64.9%, 137/211) and felt that the dogs made their holiday worse. Most respondents stated that the dogs had a low impact (one to three; 68%, 187/275) on their stay in Samoa, whilst 24% (65/275) and 8% (23/275) stated they had a medium or high impact, respectively, on their stay. No significant associations were found between gender (*p* = 0.51, Cramer’s *V* = 0.07), dog ownership (*p* = 0.09, Cramer’s *V* = 0.17), or length of stay (*p* = 0.86, Cramer’s *V* = 0.03) and whether the dogs affected the respondents’ holiday.

### 3.7. Comments

Just under half of the respondents provided additional comments (47%; 132/281). Most commonly, respondents mentioned one or more welfare issues (e.g., dog condition 26.5%; 35/132), social issues (e.g., having been chased by dogs 15.2%, 20/132) or that a change in dog control was needed (58.3%; 77/132). Some respondents indicated a connection to a person bitten by a dog or had been bitten themselves (5.4%, 8/149). A small number of the respondents also mentioned barking at night as a specific problem (4.7%, 7/149).

Number of respondents in different areas—Apia Urban Area (AUA): 82, Upolu except AUA: 94, Savaii unspecified: 50, Savaii dog free village: 17.

## 4. Discussion

It is evident from this study that free-roaming dogs in Samoa are common, have a significant effect on the holiday experience of visitors, and that this effect is perceived as being negative. However, given the nature of the data collected, analyses have focused on comparisons between discrete variables. These discrete variables increase the likelihood that statistically significant associations may be incidental. Although, given parity with similar studies, the authors consider this unlikely and the results should be viewed in light of this. 

The majority of respondents in the current study felt that the free-roaming dog population requires better control and management. The same sentiment is echoed in Farnworth *et al.* [7] who reported that more than half of local Samoans considered dogs a nuisance (64%) and that their numbers required management (77%). Similarly tourists in Samoa think better management is needed (81%) and that there are too many “stray” dogs in Samoa (67.9%) (Figure 1). Figures from these two studies can be compared to findings from a rural coastal region of Mexico, where approximately half the local residents, but only a third of tourists, thought there were too many dogs [18], or the Bahamas, where stray dogs were perceived as a nuisance by over 80% of local residents [19] but only 23% of tourists [20]. In the present location, there is less disparity between the tourists’ perceptions of dogs and those of the Samoan people.

In the current study, respondents in Apia saw more dogs (Figure 3) and perceived them as more of a problem compared to the rest of Samoa, possibly as a result of the higher population density of both people and dogs. Likewise, perceived “inappropriate” actions towards dogs were observed more often in Apia. Only 5% of properties are fenced in Samoa [7], thus increased population density could contribute to increased numbers of dogs remaining unattended and free-roaming. The perceived problem is greater in Apia because of the additional impact of dogs barking, especially at night. Barking has also been reported as a problem by local Samoans [7]. Likewise, barking and roaming have been reported as the most frequent neighborhood nuisances in New Providence, the Bahamas [20].

Only a small percentage of tourists in the current study believed that it was apparent that dogs in Samoa had owners and that Samoan dogs are like the dogs in their home country (Figure 1). Percentage of dog ownership amongst the tourists that responded was substantially lower than that reported for Samoans by Farnworth *et al.* [7], those numbers being 32.3% and 88%, respectively. Similarly, only a minority of tourists cited relational ownership of dogs as “guard dogs” as opposed to 79% of Samoan households where functional ownership “for protection” was reported [7]. These findings probably represent cultural differences between tourists’ and Samoan residents’ attitudes towards dogs and their role. Despite these cultural differences there is still evidence from both groups that management is required. It is the underlying motivations and cultural differences that may result in conflict concerning free-roaming dog population management solutions, therefore further study in this area is required.

Over two thirds of the respondents neither thought dogs in Samoa were well fed, nor, in their opinion did they look healthy, and a majority felt sorry for them (Figure 1). These findings are comparable to studies in tourist areas in other countries. In rural Mexico, half of the tourists and less than a third of the local population were concerned about dog welfare [18]. In the Bahamas, “feeling sorry” for the dogs was the main reaction of a quarter of the American tourists that had seen free-roaming dogs there and as many as two thirds of the respondents in that study thought it was cruel to allow dogs to roam [20]. In addition to the perceived reduced general health and body condition among the dogs in Samoa, a quarter of the respondents in this study had witnessed actions perpetrated against dogs by residents that they considered to be “inappropriate” (Table 1). Most reported some form of physical abuse including beating, kicking, and hitting with objects. Farnworth *et al.* [7] noted that a quarter of Samoan respondents considered it appropriate for Samoan people to inflict harm on or kill dogs. This compares to less than 10% of respondents in the present study thinking the use of violence against dogs is acceptable, with a few respondents mentioning defense or protection (Figure 1). Again such differences in opinion may result in conflict and, in the present study, those respondents who witnessed dogs being treated inappropriately were likely to report a more negative holiday experience. Ruiz-Izaguirre and Eilers [18] reported that villagers in areas frequented by tourists were more concerned about dog welfare problems than villagers in a farming village. It could be suggested from this finding that the presence of tourists and their influence may help improve the local perception of dog welfare; conversely improved local treatment of dogs may also improve the tourist experience and therefore the tourism industry.

Nearly two thirds of the respondents in the current study had felt threatened by dogs in Samoa (Table 1) and some respondents mentioned that either they or a companion had been bitten. The report of feeling threatened is substantially greater than in other studies. In the Bahamas, only 5% of tourists that saw free-roaming dogs stated they felt scared or threatened [20]. The degree of threat perceived in the present study may be realistic as there are high numbers of reported dog bites of humans in both Samoa and American Samoa [7,8].

With an acceptable spread between genders, age groups, and home countries we propose that the respondents in this study represent a reasonable sample of tourists to Samoa. However, as tourists in Samoa tend to travel to different locations around the islands, it is difficult to match the figures from different areas to “true” values of area distribution, though effort was made to sample all areas with emphasis on tourist dense areas. Tourists generally depend on the Samoan Tourism Authority and the tourism industry to provide them with information to help them be conscientious visitors [21]. Such information should include information on dog prevalence and problems in different parts of Samoa, as well as recommendations on appropriate behavior to prevent tourists exposing themselves to undue risk. According to the same study [21], tourists also believed that they had a responsibility to better understand the Samoan culture. Whether or not this results in implicit acceptance of the free-roaming dogs in Samoa or reduces tourists’ likelihood to voice a perceived need for change is unsubstantiated. However, it is likely important to provide information to both the Samoan residents and tourists in order to improve perceptions and management of free-roaming dogs. Primarily, the misalignment of opinions around dogs is presumably driven by the role of dogs in Samoa. Unlike the dogs of visitors, the majority of dogs in Samoa are used for either hunting or protection [7].

Tourists to Samoa are predominantly from New Zealand and Australia [1] and are therefore likely to have very different perspectives on dog care and ownership. As inappropriate actions towards dogs were witnessed (Table 1), and significantly reduced the tourists’ experiences of Samoa, it is likely that this attitude will also extend to inappropriate or inhumane population management techniques. In this study, all non-lethal methods of management were preferred to the lethal methods (Figure 3). Voluntary sterilization earned the greatest support with nearly 90% of respondents regarding this method as an acceptable method for dog population management.

It should be noted that a majority of the tourists surveyed in Samoa regarded themselves as “dog lovers” and felt sorry for the dogs (Figure 1) and that they disagreed strongly with inhumane management methods such as poisoning (Figure 3), which has been used in the past to control dog populations in Samoa [22]. Tourists also reported a greater level of perceived acceptability for non-lethal compared to lethal control methods (Figure 3). Therefore, support from tourists may be contingent upon the use of humane and non-lethal management wherever possible. However, most Samoans have no access to veterinary services [23], therefore increasing the frequency of access to charity clinics and funding to support free sterilization clinics might be required.

Management of the free-roaming dog population requires active participation by animal control officers and veterinarians. The limited nature of governmental provision of such services in Samoa constrains the ability to implement effective, and locally acceptable, population management measures. Risks posed to tourists and locals related to both injury and disease, are of significant concern. Likewise, the presence of free-roaming dogs is of significant concern to Samoa as they may impact negatively on the tourist trade, Samoa’s primary industry. Limitations placed on the ability of dogs to free-roam and management of currently free-roaming individuals are identified as vital components of any dog population control strategy [24]. These key points may, however not be viewed with enthusiasm in Samoa, as most dogs in the community are either free-roaming or not confined to the owners’ property [7].

Population management modeling has identified sterilization as the most efficient method to achieve sustained reductions in target populations [25]. It has also been found that sterilized free-roaming dogs in India had a higher body condition score than intact dogs in the same area [17]. Among the lethal management methods, a majority of the respondents supported humane euthanasia (Figure 2). Humane euthanasia alongside regular sterilization campaigns and adoption of free-roaming puppies and juvenile dogs to suitable owners has been shown as effective in managing the population [9]. Again, in the absence of veterinary and animal services provision, humane euthanasia techniques may not always be used in Samoa. Given that most Samoan’s consider dogs to be “one of the family” [7] it is likely that dog owners in Samoa will be open to learning about dog behavior and welfare if provided in a culturally appropriate way. In this regard it may be important to promote (and improve access to) veterinary and allied services for Samoans.

## 5. Conclusions and Implications

Free-roaming dogs are of significant concern in Samoa having an impact upon both local residents and tourists. The findings of the current study, along with previous findings of Farnworth *et al.* [7] identify a clear need for improvements in managing the free-roaming dog population, including greater provision of veterinary care. There is significant support for dog population management in the local population [7], which is also supported (with an additional call for humane management of free-roaming dogs) by tourists. Humane management may not only positively impact upon the tourism industry in Samoa, but may also improve the welfare of the free-roaming dog population and minimize both disease and injury risk for local residents. This study helps identify priorities relating to the management of free-roaming dogs and will help when allocating both national and international funding to address the welfare of free-roaming dogs in Samoa. Increased funding for veterinary care facilities, development of municipal dog management strategies, education for local schools and adults, and improved tourist information will all assist in reducing the impact (and perhaps improving the welfare) of free-roaming dogs in Samoa.

## Supplementary Files

Supplementary File 1
