# Supplementary material for: Tourists’ Perceptions of the Free-Roaming Dog Population in Samoa
Source: Animals (Basel). 2014 Sep 29;4(4):599–611. doi: 10.3390/ani4040599 (PMC4494425; doi:10.3390/ani4040599)
Supplement: Supplementary File 1 [file animals-04-00599-s001.docx]

Supplementary Information

Information for Participants

Tourist Attitudes towards and Behaviour around Dogs in Samoa

Greetings

This questionnaire will gather facts about dogs in Samoa and your attitudes and behaviour towards them. This project aims to find out what factors have an effect on tourists’ responses to dogs and opinions about their care and management.

What This Means for You

By taking time to fill out and return this questionnaire you let us know common factors which influence tourists’ opinions about dogs.

This information is not known for the Pacific region, gathering it will allow conclusions to be made. These conclusions may be used to find and get funding for educational campaigns and veterinary services.

What Will Be Done with This?

Information gathered will be put together and analysed. It will be sent to all participating organisations in New Zealand. Unitec Institute of Technology and Massey University also reserve the right to present and publish the work where appropriate.

Consent

Participation in the survey is taken to indicate informed consent.

Ethical approval for this study has been confirmed by Massey University, New Zealand.

Confidentiality

All participants will remain anonymous and the hard copy of all data collected will be placed in a password protected file, or locked cabinet, and kept for five years.

**Section One: Respondent’s Details.**

This section is to get some background data. Please answer all questions by ticking the appropriate box.

| (1) Gender of respondent | [ ] Male | [ ] Female |
| --- | --- | --- |
| (2) Age group of respondent | [ ] 0–20 | [ ] 21–40 |
|  | [ ] 41–60 | [ ] 61–80 |
|  | [ ] 80+s |  |
| (4) Do you own a dog? | [ ] Yes: pet-guard-working dog (please circle) | [ ] No |
| (5) Country of residence | _____________________ | _______________ |
| (6) How long have you been in Samoa? | _____________________ | _______________ |

**Section Two: Your Opinions on Dogs.**

Please read each of the statements below and tick relevant box.

|  | **Absolutely Agree** | **Agree** | **Neither Agree nor Disagree** | **Disagree** | **Absolutely Disagree** | **Don’t Know** |
| --- | --- | --- | --- | --- | --- | --- |
| In general dogs are dangerous animals |  |  |  |  |  |  |
| In general dogs are a nuisance |  |  |  |  |  |  |
|  | **Absolutely Agree** | **Agree** | **Neither Agree nor Disagree** | **Disagree** | **Absolutely Disagree** | **Don’t Know** |
| I am a dog lover |  |  |  |  |  |  |
| I feel safe around dogs here |  |  |  |  |  |  |
| Dogs clearly have owners here |  |  |  |  |  |  |
| Dogs here are well fed |  |  |  |  |  |  |
| These dogs are like those in my home country |  |  |  |  |  |  |
| Dogs here look healthy |  |  |  |  |  |  |
| I feel sorry for dogs here |  |  |  |  |  |  |
| Dogs are a nuisance here |  |  |  |  |  |  |
| There are too many stray dogs here |  |  |  |  |  |  |
| Dog poo should be cleaned up more often heres |  |  |  |  |  |  |
| In general dogs spread disease |  |  |  |  |  |  |
| Dogs require better management here |  |  |  |  |  |  |
| The use of violence against dogs is acceptable |  |  |  |  |  |  |

**Section Three: Experiences with and Behaviour around Dogs in Samoa.**

Please answer each question by ticking the appropriate box.

|  | Yes, Always | No, Not at All | Sometimes |
| --- | --- | --- | --- |
| Do you avoid contact with dogs here? |  |  |  |
| Have you seen free roaming dogs in Samoa? |  |  |  |
| Do dogs here beg for food? |  |  |  |
| Do you feed the dogs here? |  |  |  |
| Do you wash your hands after contact with a dog? |  |  |  |
| Do you feel dogs are friendly here? |  |  |  |
| Do you feel threatened when a dog approaches you here? |  |  |  |
| Do dogs here seem frightened and avoid human contact when approached? |  |  |  |
| Do you wash your hands before a meal? |  |  |  |
| Do you wear shoes or sandals on the beach? |  |  |  |
| Have you witnessed inappropriate behaviour against dogs in Samoa and define inappropriate |  | | |

**Section Four: Overall Experience.**

| On a scale of 1–10 how much have the dogs affected your holiday where 1 = “not at all” and  10 = “very serious impact”. | | | | | | | | | | | | | |
| --- | --- | --- | --- | --- | --- | --- | --- | --- | --- | --- | --- | --- | --- |
| Please circle: | 1 | 2 | | | 3 | 4 | 5 | | 6 | 7 | 8 | 9 | 10 |
| Was this effect POSITIVE (the dogs improved your holiday) or NEGATIVE (the dogs made your holiday worse). | | | | | | | | | | | | | |
| Please circle: | | | | Positive | | | | | | Negative | | | |
| In which part of Samoa did you spend most time during this holiday? | | | | | | | | | | | | | |
|  | | | | | | | | | | | | | |
| Do you feel free roaming dogs in this area are a problem? | | | | | | | | | | | | | |
| YES | | | | | | | | NO | | | | | |
| How would you describe the number of different dogs you saw around your accommodation?  Please circle: | | | | | | | | | | | | | |
| None | | | A small number (1–4) | | | | | Quite a lot (5–10) | | | Large numbers (11+) | | |

**Section Five: Dog Management.**

How acceptable do you consider the following methods of dog population management? Please mark the appropriate box.

|  | **Absolutely Acceptable** | **Somewhat Acceptable** | **Neither Acceptable nor Unacceptable** | **Somewhat Unacceptable** | **Absolutely Unacceptable** | **Don’t Know** |
| --- | --- | --- | --- | --- | --- | --- |
| Voluntary sterilisation |  |  |  |  |  |  |
| Compulsory sterilisation |  |  |  |  |  |  |
| Voluntary registration |  |  |  |  |  |  |
| Compulsory registration |  |  |  |  |  |  |
| Humane Euthanasia of strays |  |  |  |  |  |  |
| Humane euthanasia of unwanted puppies |  |  |  |  |  |  |
| Compulsory Collars and ID tags |  |  |  |  |  |  |
| Poisoning of strays |  |  |  |  |  |  |
| Fencing to prevent dogs wandering |  |  |  |  |  |  |

| Your Comments: |
| --- |

Location:

Thanks for filling out our survey. We hope you enjoy your holiday and have a safe trip home.
